# Skin-conformal PMN-PT ultrasonic sensor for cuffless blood pressure sensing via eutectic solder integration

**DOI:** 10.1038/s41378-025-01110-2

**Published:** 2026-01-01

**Authors:** Syed Turab Haider Zaidi, Dong Hun Kim, Muhammad Ali Shah, Young Jin Lee, Byung Chul Lee, Shin Hur

**Affiliations:** 1 Department of Bionic Machinery, Korea Institute of Machinery and Materials, Daejeon, 34103 South Korea; 2 University of Science and Technology, Daejeon, 34113 Korea; 3 Bionics Research Centers, Korea Institute of Science and Technology, Seoul, 02792 South Korea; 4 KHU-KIST Department of Converging Science and Technology, Kyung Hee University, Seoul, 02447 South Korea

**Keywords:** Engineering, Materials science

## Abstract

Wearable ultrasonic systems are emerging as promising tools for noninvasive cardiovascular monitoring, enabling the real-time assessment of vascular dynamics without the need for cuff-based measurements. However, the integration of high-performance piezoelectric materials into flexible, skin-conformal arrays poses challenges in terms of mechanical stretchability, acoustic fidelity, and scalability. In this study, we present a flexible 5 × 4 ultrasonic transducer array (UTA) based on 1-3 lead magnesium niobate-lead titanate (PMN-PT) composite elements for continuous blood pressure monitoring. To achieve reliable integration, we employed a dual-sided eutectic solder bonding method using an Sn-Bi alloy to ensure low-temperature attachment without depolarization. The fabricated UTA operates at a center frequency of 6.0 MHz with an acceptance angle of 45°, enabling acoustic signal acquisition under varying angular conditions. Time-of-flight simulations and in vitro testing of vascular phantoms demonstrated the accurate tracking of vessel diameters and the estimation of real-time blood pressure. The UTA achieved systolic and diastolic pressure measurements within 4 mmHg of those of a commercial reference sensor. These results highlight the feasibility of scalable and flexible ultrasound systems for wearable hemodynamic sensors, suggesting their potential for next-generation point-of-care diagnostics.

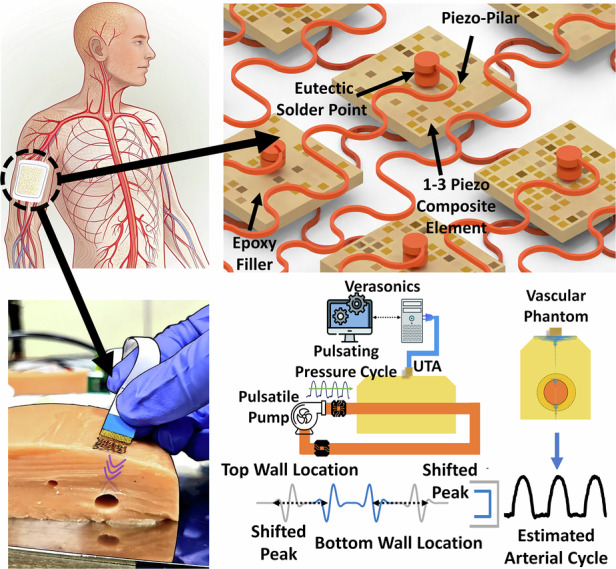

## Introduction

Continuous blood pressure (BP) monitoring is a critical component of advancing cardiovascular health management and provides essential information on vascular dynamics that is not accessible through intermittent, cuff-based measurements. Accurate and real-time BP tracking allows for early detection of vascular abnormalities, dynamic assessment of hemodynamic changes, and improved management of conditions such as heart disease and stroke. Traditional cuff-based BP devices, which are widely used in clinical and home environments, are bulky, non-continuous, and uncomfortable for long-term use. Moreover, they are unsuitable for ambulatory or wearable applications that require user comfort, continuous data acquisition, and motion tolerance^1–3^. To address these limitations, a variety of cuffless wearable BP monitoring approaches have been explored, including optical photoplethysmography (PPG), tonometry, and pulse transit time (PTT)-based systems^4–6^. Although these technologies offer noninvasive and portable solutions, they are highly sensitive to confounding factors such as skin tone, motion artifacts, ambient light, and superficial vascular variations^7,8^. Furthermore, their performance is often compromised when used for deeper vascular monitoring, which limits their clinical reliability and accuracy. However, ultrasound has emerged as a promising modality for continuous BP-monitoring owing to its capabilities, such as deeper tissue penetration, real-time imaging, and independence from optical or superficial signal fluctuations^9,10^. Despite its potential, wearable ultrasound technology faces substantial design and fabrication challenges. Conventional ultrasound systems are typically rigid and rely on handheld probes or stationary platforms, which limits their applicability to wearable, skin-conformal use. Recent research has explored the integration of ultrasonic transducer arrays onto flexible substrates, enabling wearable ultrasound systems capable of continuous vascular monitoring and reconstructing blood pressure waveforms with high temporal resolution^11–15^. However, these approaches encounter several technical barriers, particularly those related to the fabrication and integration of high-performance piezoelectric materials into flexible platforms.

Lead-based piezoelectric ceramics like Pb(Zr,Ti)O₃ (PZT) have long been the standard due to their high piezoelectric coefficients, strong electromechanical coupling, and simple processing, though concerns over environmental and health impacts of lead have driven the development of lead-free alternatives such as (K,Na)NbO₃ (KNN)^16–18^. While KNN ceramics have shown notable performance improvements through compositional tuning and texturing, they generally remain below PZT in terms of absolute piezoelectric coefficients and operational stability, though recent advances are narrowing this gap^19,20^. More advanced still, lead magnesium niobate-lead titanate (PMN-PT) single crystals offer superior d₃₃ coefficients, higher electromechanical coupling, greater strain response, and better stability under high fields and temperatures^21,22^. Unlike isotropic polycrystalline PZT, the anisotropic single-crystal nature of PMN-PT allows directional optimization, making it ideal for demanding applications like medical ultrasound and precision actuators, although its higher cost and mechanical fragility remain limiting factors compared to PZT. Furthermore, 1-3 piezoelectric composites are better for required vibration in the thickness direction mainly because their structure enhances thickness-mode electromechanical coupling and reduces unwanted mode interference, leading to higher efficiency and bandwidth^21–23^.

One key challenge in developing flexible ultrasonic transducer arrays is the fabrication of reliable, low-resistance electrical interconnections between individual piezoelectric elements and flexible electrodes. Previous studies employed 3D-printed stretchable electrodes to achieve mechanical flexibility; however, these systems are often limited by poor resolution, reduced electrical conductivity, and mechanical instability under repeated bending or stretching^24,25^. Recent developments demonstrated flexible silicon-nanocolumn-based flexible electrodes and silver-nanowire-based stretchable electrodes, which enabled advanced high-resolution, disposable biomedical imaging through enhanced acoustic coupling and device conformity^26,27^. Other approaches have used conventional soldering techniques for electrical interconnection. However, these integration methods typically require processing temperatures exceeding 200 °C, which can depolarize or damage high-performance piezoelectric materials, such as lead magnesium niobate-lead titanate (PMN-PT)^28–30^. This depolarization results in degraded piezoelectric coefficients, reduced electromechanical coupling, and an overall loss of transducer performance. To overcome these challenges, it is essential to develop low-temperature integration methods that preserve the functional integrity of piezoelectric elements while ensuring reliable mechanical and electrical bonding on flexible substrates. In this study, we present the design, fabrication, and experimental validation of a flexible 5 × 4 ultrasonic transducer array (UTA) based on PMN-PT 1-3 composite elements. The UTA is specifically developed for continuous BP monitoring in wearable applications. In addition, our array is fabricated using a dual-sided eutectic bonding process with the Sn-Bi alloy, enabling low-temperature assembly below 150 °C. This approach prevents depolarization of the PMN-PT elements and provides mechanical and electrical interconnections that can withstand bending and skin-conformal deformations.

The designed UTA operates at a center frequency of 6.0 MHz and features a 45° acceptance angle, making it suitable for vascular interrogation under dynamic conditions. To evaluate the system performance, time-of-flight (TOF) simulations were conducted to estimate the vessel wall displacements and pressure changes. Furthermore, in vitro experiments using a virtual blood vessel phantom validated the ability of the array to detect changes in vessel diameter and estimate continuous systolic and diastolic BP with high accuracy.

## Design and simulation

### Structural design and sensing principle

**Fig. 1:**
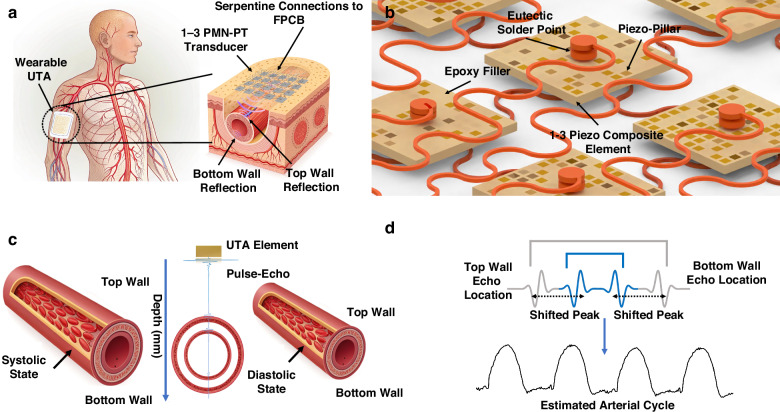
Conceptual overview and functional workflow of the PMN-PT UTA system. **a** Anatomical illustration showing wearable UTA placed on the upper arm for vascular monitoring. **b** Enlarged view of the PMN-PT 1-3 transducer bonding with serpentine flexible interconnects and eutectic solder. **c** Pulse-echo behavior during systole and diastole in a vessel phantom model. **d** RF echo analysis enables the extraction of vessel wall motion and the reconstruction of blood pressure waveforms

**Fig. 2:**
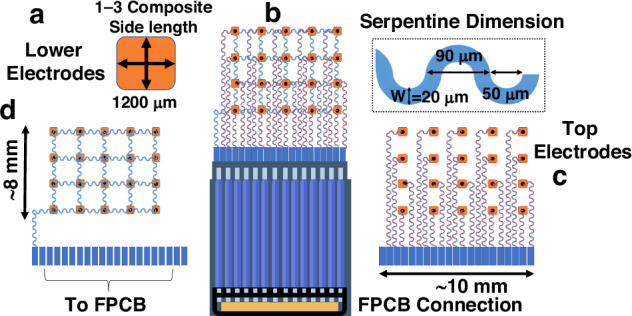
Schematic design of the UTA. **a** Individual piezoelectric transducer. **b** Schematic of the UTA array. **c** Layout of the top electrodes. **d** Common lower electrode configuration

A flexible ultrasonic transducer array (UTA) was developed for continuous noninvasive monitoring of blood pressure waveforms. The UTA shown in Fig. 1a conforms to the skin surface and transmits high-frequency ultrasound at 6.0 MHz to examine the superficial vasculature. As shown in Fig. 1c, d, echoes reflected from the vessel walls are analyzed to track diameter changes during pulsatile blood flow, enabling the reconstruction of the arterial pressure cycle. This configuration supports both static vessel localization and real-time blood pressure estimation. Figure 2 shows a schematic of the designed array. The design and fabrication processes are explained in detail in the following subsections. Validation of the UTA is conducted in two phases: first, radiofrequency (RF)-based imaging of embedded vessel phantoms is used to determine spatial localization and vessel diameter, and second, in vitro testing under pulsatile flow conditions is performed to capture dynamic wall displacement and reconstruct systolic and diastolic pressure waveforms.

**Fig. 3:**
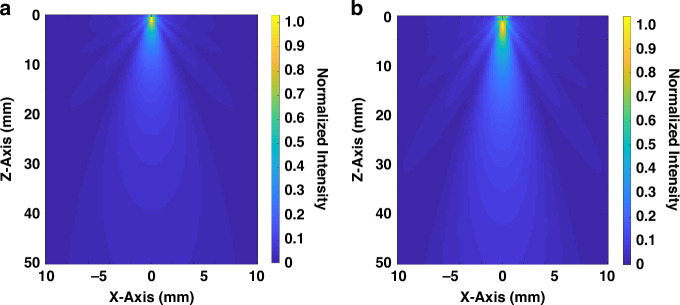
Acoustic field simulation for 1-3 piezoelectric composites with different sizes. **a** 0.9 mm × 0.9 mm. **b** 1.2 mm × 1.2 mm implemented in TAC_GUI toolbox MATLAB

To define the optimal transducer aperture for vascular targeting, acoustic field simulations are conducted using the TAC_GUI toolbox in MATLAB^31^. Square PMN-PT 1-3 composite elements with side lengths of 0.9 mm and 1.2 mm are modeled to assess their penetration depth, lateral confinement, and directivity. As shown in Fig. 3, the 1.2 mm element design achieves up to 40 mm penetration in the axial (z) direction, compared to 35 mm for the 0.9 mm aperture. At a normalized pressure intensity of 0.1, the 0.9 mm element exhibits side lobe levels ranging from –22 dB to –27 dB, whereas the 1.2 mm element shows slightly elevated side lobe levels at –20 dB to –25 dB. These results indicate that a larger aperture provides superior beam intensity at a target depth of 7.3 mm, making it suitable for accessing superficial arteries. While a higher side lobe energy represents a trade-off, the resulting gain in spatial resolution and signal-to-noise ratio justifies the selection. These simulation findings indicate that the final aperture design enables the formation of a full acoustic window across a vessel diameter of approximately 2.7 mm.

The final 5 × 4 UTA layout is developed to achieve sufficient lateral coverage of the vascular region while maintaining the mechanical flexibility and beamforming capability. The selected element size, determined by favorable beam characteristics, provides an adequate penetration depth and low side lobe intensity, both of which are essential for accurately targeting vessels at a depth of 7.3 mm. To ensure lateral coverage of the target vessel diameter, five elements are arranged in a linear 1 × 5 subarray to form an effective acoustic aperture. This subarray is then vertically replicated to construct a full 5 × 4 matrix, yielding 20 individually addressable elements optimized for spatial resolution and mechanical conformity. The configuration enhances sampling across both the lateral and elevational planes while preserving the structural compliance necessary for wearable applications. The electrical design of the array features individually addressable top electrodes and a shared bottom ground plane, simplifying interconnection. PMN-PT was selected as the piezoelectric material due to its high electromechanical coupling coefficient and acoustic impedance, measured at approximately 16.5 MRayl for the 1-3 composite configuration. The material characteristics of PMN-PT improve the acoustic energy transmission across tissue boundaries and enhance detection sensitivity. Combined, these design considerations support a robust, high-acoustic transmission–reception UTA engineered for continuous and noninvasive blood pressure monitoring in a wearable form.

### Multiphysics simulation

**Fig. 4:**
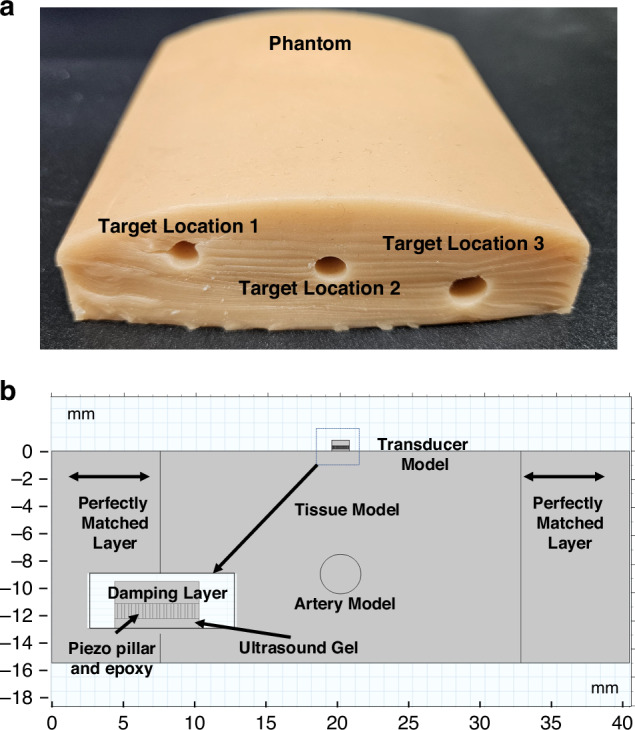
Physical layout of the vascular phantom and simulation model. **a** Location of the vessels inside the phantom. **b** Schematic of the COMSOL simulation model with a zoomed view of the transducer

To evaluate the acoustic performance of the proposed transducer, a multiphysics finite element model is developed using COMSOL Multiphysics. The simulation domain includes a multilayer structure composed of an epidermal skin based on a virtual vascular phantom (model VATA-0705), a perfectly matched layer (PML) for acoustic boundary absorption, and the PMN-PT 1-3 composite element. This model is used to simulate the propagation and reflection of ultrasound waves at soft tissue interfaces. In addition, the simulation is performed in two steps. First, the wave propagation from the transducer to the proximal vessel wall and the associated reflection are analyzed. Second, the subsequent reflection from the distal vessel wall and its return to the transducer are simulated^32,33^. The geometric parameters of both the phantom vessel and the transducer are defined according to the physical design shown in Fig. 4a, b. A virtual coupling layer, modeled as an ultrasound gel layer, is placed between the UTA and phantom surface, and the PML is used to eliminate spurious boundary reflections.

**Fig. 5:**
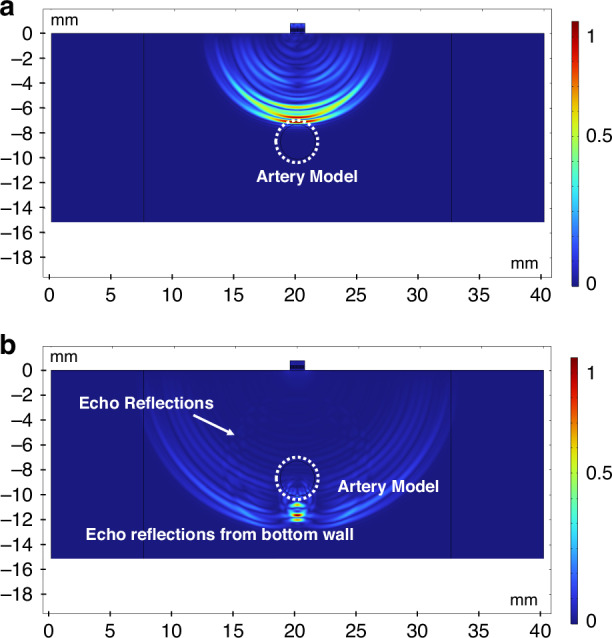
Transmission of the ultrasound pulse inside the phantom material with observable echo pulses at the time sample. **a** At *T* = 5.4 μs acoustic wave reflecting from the top wall. **b** At *T* = 9.4 μs acoustic wave reflecting from the bottom wall

**Fig. 6:**
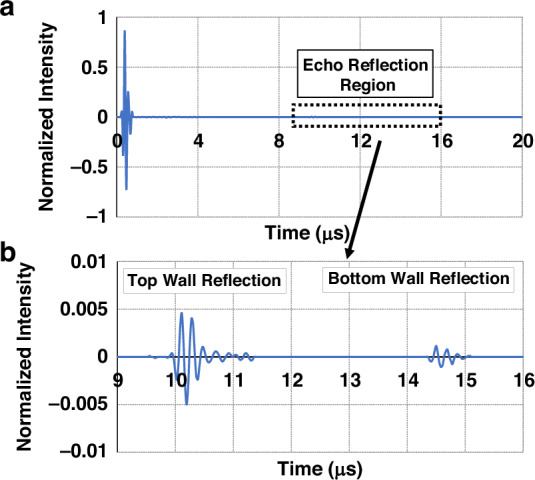
Results of measuring the ultrasonic pulse-echo signals incident on the virtual vessel phantom and the reflected wave signals from the virtual vessel. **a** Complete pulse-echo signal measured across time. **b** Pulse-echo signal portion showing reflected wave from the vessel walls

The mesh resolution is determined based on the acoustic wavelength (*λ* = *v / f*) in soft tissue, where *v* and *f* are the material acoustic velocity and operating frequency, assuming an acoustic impedance of 1.71 MRayl and an operating frequency *f* of 6.0 MHz, to ensure numerical convergence and accuracy. The acoustic impedance of the blood-vessel phantom is set to 1.65 MRayl. For experimental benchmarking, vessel diameter and depth measurements are performed using a commercial ultrasound probe (L12-4V, Philips), and representative B-mode images are provided in Fig. S1^34^. The simulated pressure-field distributions within the tissue phantom are shown in Fig. 5. At 5.4 μs, the acoustic wave reflects from the proximal (upper) wall of the vessel, and at 14.2 μs, it reflects from the distal (lower) wall. These reflections are captured by the transducer model and used to estimate the vessel depth and diameter. Figure 6 presents the time-domain ultrasound pulse-echo response derived from the finite-element model. The input excitation signal was a 6.0 MHz pulse-echo signal with a 20 V amplitude, and the input waveform is shown in Fig. S2. The resulting echo signals exhibit distinct envelopes, each corresponding to an interface with different acoustic impedances. The first peak is attributed to the surface interface, whereas the subsequent reflections correspond to the proximal and distal vessel walls. The received waveforms indicate reflection arrival times at 10.2 μs (proximal wall) and 14.6 μs (distal wall), suggesting a vessel diameter of 2.7 mm based on the time-of-flight (*TOF* = 2 × *Distance*/*Speed*) principle. Additional vessel configurations for target locations 2 and 3 were simulated using the same methodology. Table 1 summarizes the TOF-derived data for the three target vessel locations.

**Table 1:** Pulse-echo time calculation for the static diameter of the target vessel

Phantom targets	Depth (mm)	Diameter (mm)	Echo 1 time (μs)	Echo 2 time (μs)
Location 1	7.3	2.7	10.22	14.6
Location 2	13.8	3.8	19.7	25.85
Location 3	16.5	6.6	23.91	33.69

The point distance is calculated using Time of Flight = 2 *ct*, where *c* = ~1400 m/s is the acoustic speed of sound and *t* is the echo time (µs). The mesh size of the FEM model is maintained at λ/4

### Fabrication process

**Fig. 7:**
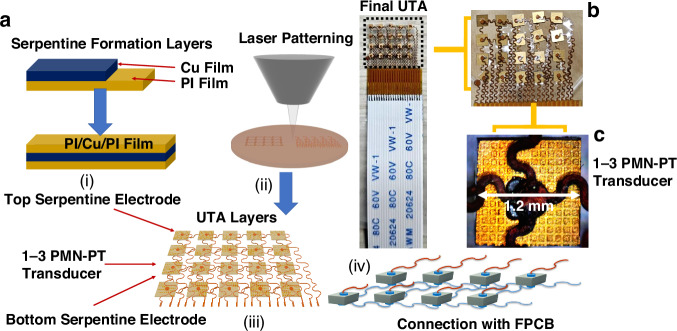
Fabricating and bonding of the UTA. **a** Overview of the fabrication process. (i) Spin coating of PI on Cu sheet, deposition of Cu sheet, and an additional PI coating. (ii) Laser patterning for the electrode arrangement. (iii) Individual layers showing both electrodes and the transducer element. (iv) Integration of the electrode layers with the composite (commercially manufactured iBULe Photonics, Korea) and FPCB. **b** Formation of the final array with FPCB connection. **c** An individual UTA element with bonding

The fabrication process of the 5 × 4 UTA, as shown in Fig. 7, begins with the preparation of flexible electrodes on a 100 μm-thick polyimide (PI) substrate, which is selected for its balance of flexibility and mechanical stability. An 18 μm-thick copper layer is electroplated onto the PI substrate, followed by the deposition of 5 μm of nickel and 0.05 μm of gold to enhance solderability. The electrode patterns are defined using standard photolithography and etching techniques. The 1-3 piezocomposite blocks are diced to a side length of 1.2 mm × 1.2 mm, aligned onto the prepared contact pads, and bonded to the electrodes.

A key innovation in this work is the implementation of a dual-sided bonding process using low-temperature Sn-Bi eutectic solder (melting point approximately 138 °C), which prevents depolarization by maintaining bonding temperatures below the Curie temperature of PMN-PT (approximately 170 °C). In this process, the bottom electrodes are bonded via conventional hotplate reflow at ≤145 °C to establish secure electrical contact across the ground plane. For the top electrodes, a localized hot-needle reflow technique is employed, wherein a heated needle is applied directly over the solder joint for ≤5 s. This approach restricts thermal diffusion to the surrounding areas, enabling precise solder reflow while minimizing the thermal stress on adjacent elements and preserving the piezoelectric polarization. In addition, the eutectic Sn-Bi joints exhibit stable bonding with Cu pillar and ENIG finishes, showing distinct intermetallic compound formation (Cu₆Sn₅ and Ni₃Sn₄) and demonstrating mechanical reliability under shear, thermal shock, and cyclic bending tests. Failures were primarily initiated at the solder/IMC interfaces, with lifetimes strongly dependent on bonding force and joint height, thereby confirming the suitability of Sn-Bi solder bumps for flexible electronic applications^35^. Drawing on insights from the fabrication process, the eutectic solder bonding technique also enhances the scalability of the UTA, supporting its deployment in large-scale applications, such as photoacoustic imaging, and precise small-scale applications, such as arteriole diagnostics. The dual-layer interconnect scheme supports a matrix configuration with a shared bottom–ground plane and individually addressable top electrodes. Laser micromachining is employed to define the final array geometry and form the flexible serpentine traces. Conductive silver paste is used to secure each piezoelectric element and establish a connection to the flexible circuit. The completed array is subsequently encapsulated with a parylene-C coating to ensure waterproofing and biocompatibility for wearable applications. The microstructure of the PMN-PT 1-3 composite was further verified through scanning electron microscopy (SEM). Representative SEM images of the composite surface and cross-section are provided in the Supplementary Information (Fig. S3), confirming 34% uniform ceramic pillar distribution and 66% complete epoxy infiltration. These observations validate the integrity of the dicing–filling process and support the high piezoelectric performance reported in the results section. The flexible array was fabricated on a 100 µm polyimide substrate and encapsulated with a parylene-C layer to ensure both mechanical conformity and biocompatibility for skin contact. These materials are widely used in biomedical electronics due to their inertness and safety, suggesting that the device can be comfortably worn for extended periods. Additionally, the total device thickness (<0.5 mm) and weight (<1 g) minimize skin loading.

## Results and discussion

To evaluate the performance of the flexible UTA, a comprehensive characterization encompassing electrical impedance measurements, transmission directivity, receiving angle, and acoustic pulse-echo testing was conducted. These assessments established the transmission depth, acceptance angle, and ultrasonic signal response prior to vascular phantom validation of the array. This section presents both the intrinsic device properties and the functional output of the fabricated array under controlled laboratory conditions.

### Electrical characterization

**Fig. 8:**
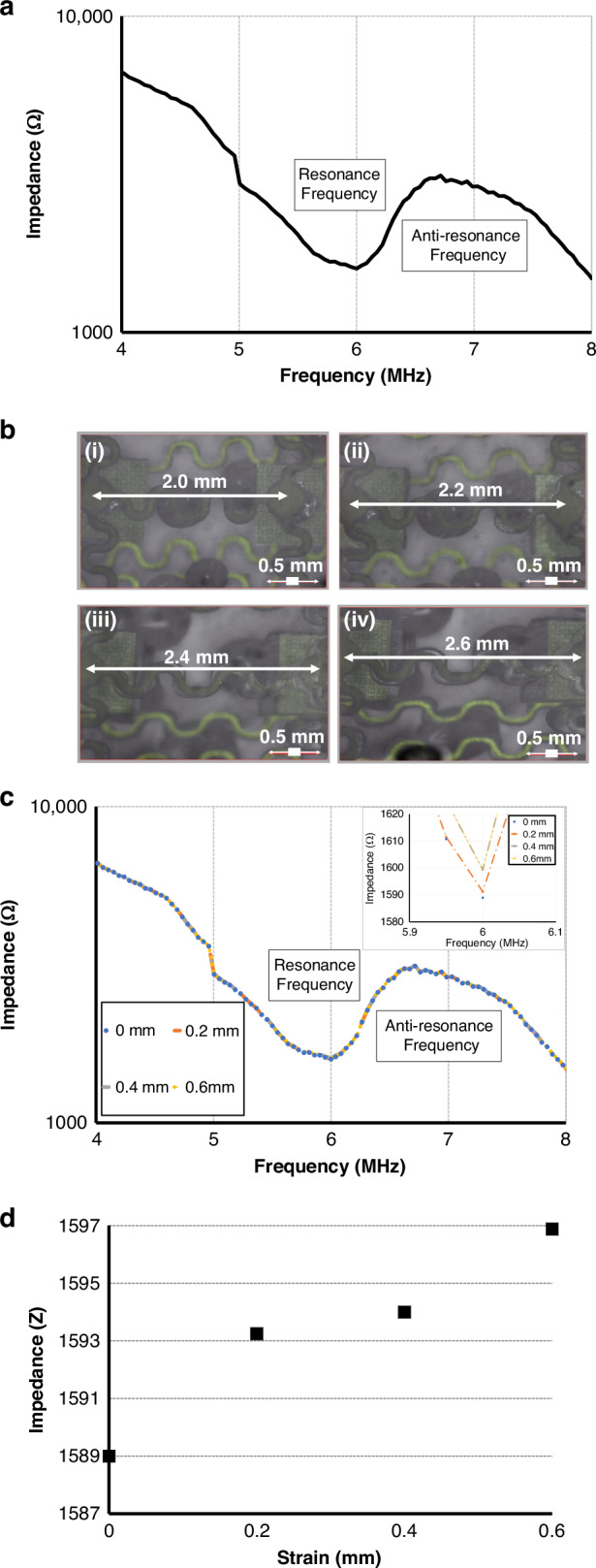
Impedance characterization of the flexible UTA under mechanical strain. **a** Impedance response of transducer element inside the UTA, **b** Demonstration of stretchability with *tensile displacement* upto 2.6 mm, **c** Measurement of electrical impedance at each strain load, **d** Comparison of impedance variation with applied *tensile displacement* load at 6 MHz operating frequency

Electrical impedance spectroscopy was performed to evaluate the resonance behavior and electromechanical coupling of the individual PMN-PT 1-3 composite elements within the 5 × 4 array. Measurements were taken using an impedance analyzer (Agilent 4294A, 20 Hz–110 MHz) with a calibrated test fixture. Each element was individually contacted, and measurements were obtained in a dry environment at room temperature. A precise resonant frequency (*f*r) of approximately 6.0 MHz and an anti-resonant frequency (*f*a) of approximately 6.6 MHz were observed across the UTA elements shown in Fig. 8a. Additional properties of the 1-3 piezocomposite are summarized in Table 2.

**Table 2:** Properties of the PMN-PT transducer element

Parameter	Design values
Side Length (μm^2^)	1200 × 1200
Thickness (mm)	0.219
Pitch (mm)	2.0
Fr (MHz)	6.0
Fa (MHz)	6.6
ε_33_T/ε0	4842
d_ij_ ×10⁻¹² C/N	d_33_ = 1282
S^E^_ij_ ×10⁻¹² C/N	S^E^_33_ = 47
Trt °C	95
Ec KV/cm	2
*Density kg/m³*	8080

To further confirm robustness, the device was subjected to incremental tensile stretching up to 130% strain, during which the inter-element spacing increased from 2.00 mm to 2.60 mm without delamination or solder joint fracture. Similar observations were made with our prior work on flexible PMN-PT ultrasonic transducer arrays, where tensile displacement up to 130% demonstrated reliable electrical continuity and mechanical stability of the serpentine electrodes^36^.

Under tensile displacement loading, the resonance frequency and impedance spectra remained stable, confirming electromechanical integrity under repeated deformation. Independent serpentine electrode tests showed elastic recovery up to ~135% strain, with only the polymer encapsulation exhibiting a tearing effect beyond 2.7 mm, while electrodes and solder joints remained functional. The strain results and corresponding impedance results are highlighted in Fig. 8b, c. In addition, the linear actuator rig setup and tensile displacement on the serpentine are shown in Fig. S4.

### Acoustic characterization

**Fig. 9:**
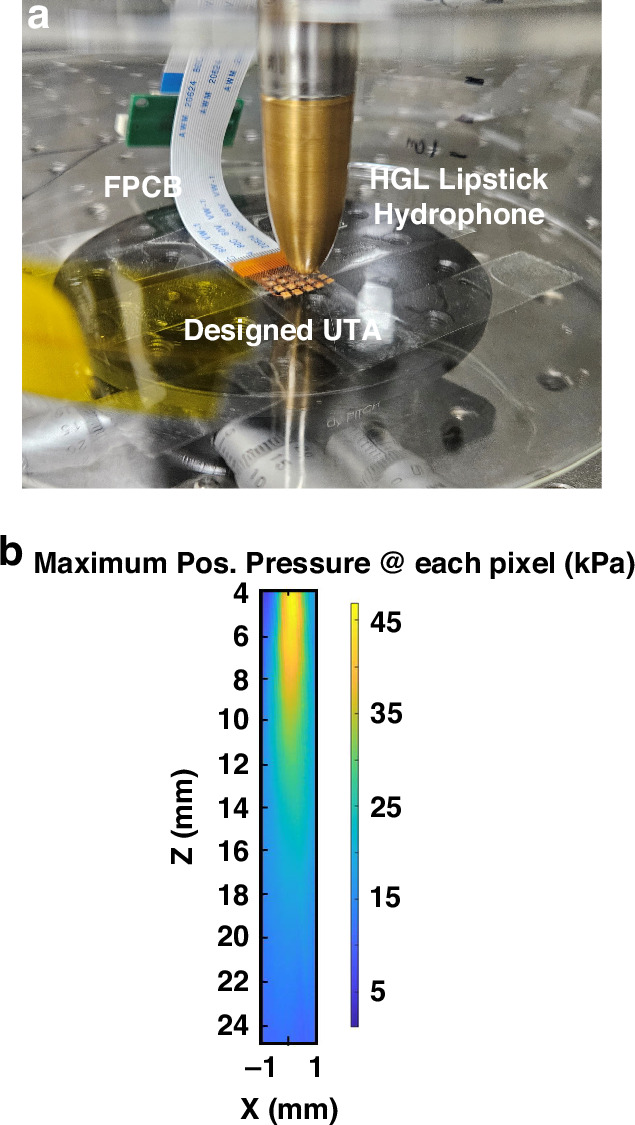
Measuring the acoustic pressure. **a** Setup for measuring acoustic transmission directivity and sensitivity. **b** Acoustic characterization results for the beam spread around the *Z*-axis direction

Following the electrical characterization, the acoustic performance of the UTA was evaluated, focusing on the transmission sensitivity, receiving sensitivity, and ultrasonic beam directivity. Transmission sensitivity, defined as the electrical input required to achieve a desired sound pressure level (SPL), was assessed by applying a sinusoidal signal, 10sin (2π*f*t) V, where *f* denotes the operating frequency, to an individual UTA element. The resulting acoustic pressure was measured using a calibrated Golden Lipstick hydrophone (HGL-0200, Onda, Sunnyvale, CA, USA) mounted on a 3D scanning platform, as shown in Fig. 9, with measurements taken at distances of up to 25 mm. The hydrophone signals were sampled at 200 MHz and displayed using an oscilloscope (MSO-X 4154 A, Keysight, CO, USA). The maximum acoustic pressure (approximately 45 kPa) was observed at approximately 6 mm, decreasing by approximately 90% at 25 mm. By increasing the driving voltage, the detectable depth was extended to 30 mm, as explored in subsequent experiments.

In addition, Fig. S5 shows the acoustic beam directivity measured using the mounted hydrophone. The beam intensity remains relatively constant from the center (±0.6 mm) to the boundary, decreasing by 13% of the peak intensity. Beyond this, the intensity gradually decreases, reaching up to 1.0 mm–1.3 mm. A further increase in the pitch distance resulted in a slight increase in intensity, which was likely influenced by the mechanical coupling from adjacent elements.

**Fig. 10:**
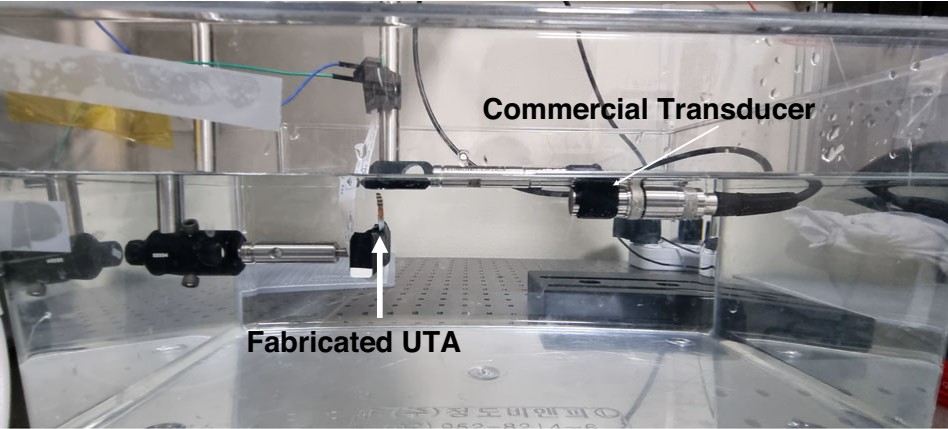
Experiment setup of using the transducer array as a receiver

**Fig. 11:**
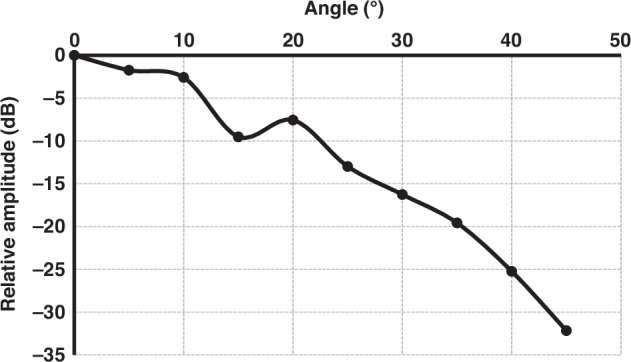
Receiving beam directivity of the transducer element

Next, the receiving sensitivity was evaluated. A higher acceptance angle indicates the ability of the transducer to effectively receive incoming acoustic signals. A calibrated A309S Olympus transducer, positioned 110 mm from the UTA, as shown in Fig. 10, generated a 6.0 MHz ultrasound beam directed at the UTA element. The received signal intensity was measured as the UTA element rotated. This rotation characterizes the receiving-beam directivity and acceptance angle. A 9-DOF IMU sensor (ST LSM6DSV + AKM AK09918 Magnetometer) mounted on the rotating platform was used to measure the yaw rotation. The received beam intensity versus the yaw rotation is shown in Fig. 11. It is evident that a 15° rotation resulted in a decrease of approximately −6 dB in signal intensity, whereas a 45° rotation resulted in a decrease of approximately −30 dB. Thus, the transducer exhibited a measured receiving acceptance angle of approximately 45°, as confirmed through this experiment, allowing effective signal acquisition even under angular misalignment, which is an essential feature of wearable configurations. The slight variations in the signal intensity with rotation may be due to the packing and arrangement of the piezoelectric elements within the UTA. Thus, we can state that an individual transducer has a high acceptance angle that can be used to align the UTA on the target specimen accurately.

**Fig. 12:**
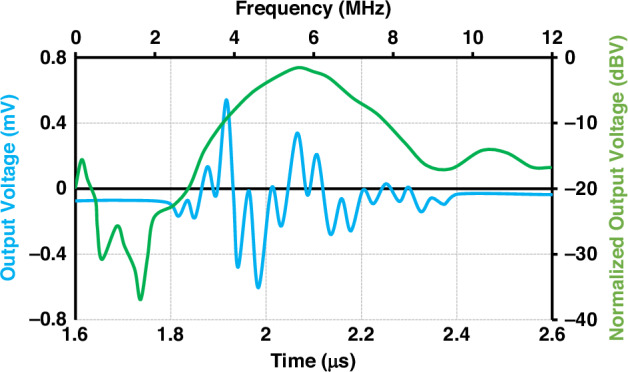
Pulse-echo performance test results of a single piezoelectric element

Finally, Fig. 12 presents the results of the pulse-echo test conducted on an individual piezoelectric transducer element within the array. The characterization demonstrated the time-domain response and frequency bandwidth of the UTA elements. The measured center frequency was approximately 6.0 MHz, with a -3 dB fractional bandwidth of 38%. This performance was achieved without the use of acoustic matching or backing layers, thereby simplifying the fabrication process while preserving sufficient signal fidelity. Furthermore, based on the applied spatial pulse, the axial resolution is approximately 0.3 mm–0.4 mm in soft tissue, which is sufficient to detect small changes in diameter.

### Blood pressure measurement based on a vascular phantom

To evaluate the dynamic sensing capability of the fabricated flexible UTA under physiologically relevant conditions, blood pressure monitoring experiments were performed using a commercial tissue-mimicking vascular phantom (VATA-0705) and the Verasonics Vantage ultrasound research platform (Verasonics Inc., USA)^37^. This system was selected because of its advanced programmable architecture and direct access to raw radio-frequency (RF) data, enabling both real-time visualization and custom signal processing.

Customized MATLAB scripts were employed in Verasonics to control the imaging sequence, allowing dynamic optimization of the transmission parameters and spatial resolution. The flexible UTA was aligned with the embedded vessel region within the phantom to ensure that the central array elements interfaced directly with the target vessel. Ultrasound gel was applied at the interface to enhance the acoustic coupling.

**Fig. 13:**
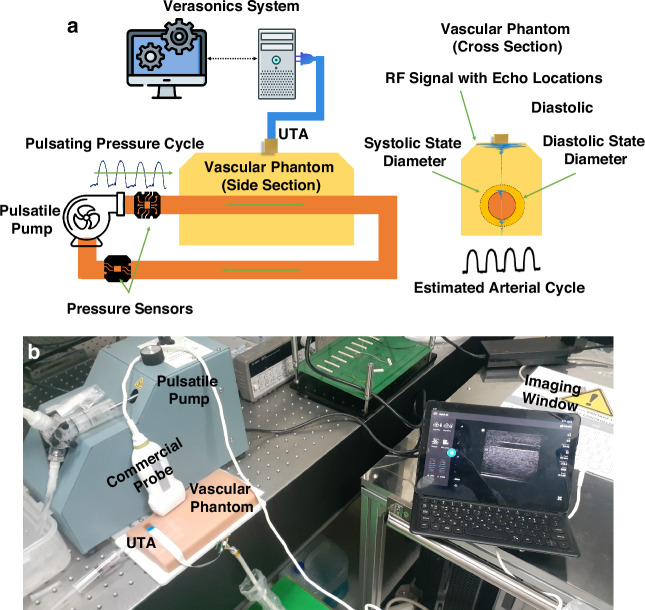
Blood pressure monitoring experiments. **a** Schematic of the experimental setup. **b** Phantom blood pressure flow with a commercial L12-4V probe along with the UTA

As depicted in Fig. 13a, b, the Verasonics system transmitted push pulses through the UTA elements along the lateral centerline of the virtual vessel and captured the RF echoes reflected from the anterior and posterior vessel walls. A transmission voltage of 20 V was applied for all the measurements. Subsequent analysis of the RF data enabled time-resolved displacement tracking in both axial (z) and lateral (x) directions. These displacements were used to characterize the vessel wall motion during pulsatile flow, forming the basis for estimating the corresponding systolic and diastolic pressure waveforms.

To assess the spatial imaging and dimensional-sensing capabilities of the fabricated UTA, RF-based axial scanning was employed to estimate the vessel diameter within the vascular phantom. Each transducer element sequentially transmitted a 6.0 MHz, 1-cycle pulse and recorded the reflected signals from the internal interfaces. The RF signal representing the pulse-echo profile was extracted to visualize the echo locations. The axial positions of the upper and lower vessel walls were identified by detecting peak reflections in the envelope. The vessel diameter was computed using the relation $D=\frac{1}{2}\cdot v\cdot \varDelta t$. Here, *Δt* corresponds to the measured time-of-flight between successive echo peaks corresponding to the anterior and posterior vessel walls. Figure S1 illustrates the target vessel location and axial depth from an average of 7.3 mm to an average of 10.0 mm, with a nominal diameter of 2.7 mm. To validate the UTA-based measurements, a commercial linear array transducer (Philips L12-4V) was used to obtain comparative ultrasound scans at the three phantom positions. A pressure sensor (Model 33A-005G-210) positioned adjacent to the phantom inlet served as a reference for the vessel pressurization during dynamic testing (Fig. S6). Figure S7 shows a typical pulse-echo response from a single UTA element. Minor reverberations were observed following the primary echo owing to the absence of a backing layer. Nonetheless, strong and distinguishable peaks enabled reliable wall detection. RF data were acquired from all 20 elements, and envelope profiles were used to estimate the diameters across the three locations. For target site 1, located at a depth of 7.3 mm, the calculated diameter averaged 2.6 mm (actual: 2.7 mm). At sites 2 and 3, located deeper, the measured diameters were 3.7 mm and 6.5 mm, respectively, compared with the respective actual values of 3.8 mm and 6.6 mm.

Table 3 presents a quantitative comparison between the UTA-based measurements and commercial probe measurements. Additionally, B-mode imaging using the Verasonics processing algorithm was applied to visualize the UTA performance at Target Vessel #1, as shown in Figs. S9a and S10, respectively, with a clear identification of the vessel walls and phantom boundary location. The vessel wall interfaces were resolved, confirming the localization accuracy of UTA.

**Table 3:** Results of the static diameter measurement of the virtual blood vessel

Target vessel	Depth of the vessel (mm)^a^	Diameter of the vessel (mm)^a^	UTA measurement (mm)^b^
#1	7.3	2.7	2.6 ± 3.8%
#2	13.8	3.8	3.7 ± 2.6%
#3	16.5	6.6	6.5 ± 1.51%

^a^Diameter measured using a commercial probe

^b^Average value of five measurements per element

To validate the capability of the developed flexible UTA for noninvasive blood pressure monitoring, we conducted pulsatile flow experiments on a vascular phantom system designed to replicate physiological conditions. A peristaltic pump (Harvard Apparatus, USA) generated a periodic flow waveform at 80 strokes per minute with a 9.0 mL stroke volume, delivering pressure cycles analogous to the human cardiac rhythm (Fig. S6). The phantom vessel was connected in series to a sealed fluidic loop, and both the UTA and commercial reference pressure sensor (Model 33A-005G-210) were positioned at a fixed measurement site (Fig. 13a). From the pressure sensor a ratiometric output obtained at 5 V excitation spans from 0.5 to 4.5 V, which is then mapped to corresponding pressure values based on the equation model, ${{\rm{V}}}_{{\rm{o}}}=\frac{0.8{V}}{{P}_{r}}{P}_{i}+0.5.$ Here V_o_ is the output voltage, *P*_*r*_ is the range of pressure here maximum range is 5 Psi or 51.7 mmHg, ${P}_{i}$ is the input pressure. Furthermore, the systolic and diastolic mapping is obtained based on the minimum and maximum values.

The Verasonics Vantage system was employed for high-frame-rate signal acquisition with a pulse repetition frequency (PRF) of 115 Hz, selected to balance the axial resolution and frame density within the target vessel depth for target vessel #1. The echo signals from the transducer elements were captured and post-processed in MATLAB to extract the real-time axial displacement of the vessel walls. The peak positions of the pulse-echo waveforms were tracked across frames, enabling temporal reconstruction of diameter variations during each cardiac cycle. Figure S8 presents the RF-scans obtained from a single UTA element at different frames to highlight the variation within the vessel wall locations.

**Fig. 14:**
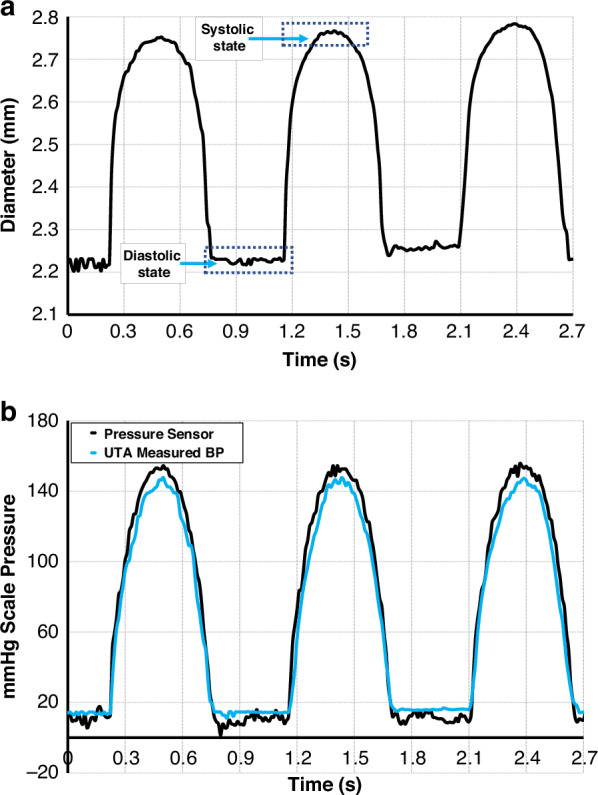
Measurements of the pulsating diameters. **a** From the UTA element. **b** Comparison of the blood pressure measurement between the transducer element and the commercial pressure sensor

Using the information of the vessel walls from both Fig. S8 and RF-scans from Fig. S9a, we observed a dynamic diameter range ranging from 2.20 ± 0.05 mm as the peak diastole to 2.76 ± 0.02 mm as the peak systole, yielding a pulsation amplitude of approximately 0.56 mm. The pulsation diameters are shown in Fig. 14a. This range of diameters was translated into pressure waveforms using a previously validated exponential model^38^. The derived relationship is based on empirical observations of the nonlinear elastic behavior of arterial walls, where pressure increases exponentially with vessel distension, defined as follows:

1 $${\rm{P}}({\rm{t}})={{\rm{P}}}_{0}{{\rm{e}}}^{\gamma A(t)}$$

Where *P*_*0*_ and *γ* are constants. This equation estimates the instantaneous arterial pressure *P(t)* from the cross-sectional area *A(t)* of the vessel. It is assumed an exponential pressure-area relationship, where *Pd* is the diastolic pressure, *A*_*d*_ is the diastolic cross-sectional area, and *α* is the rigidity coefficient capturing the stiffness of the vessel.

2 $$p\left(t\right)={p}_{d}{e}^{\frac{{\text{ln}}(\frac{{p}_{d}}{{p}_{s}})}{{A}_{d}-{A}_{s}}\left(A\left(t\right)-{A}_{d}\right)}$$

This model is particularly suitable for compliant phantom vessels where the mechanical behavior is repeatable, and the influence of viscoelastic damping is minimal. Similarly, *P*_*d*_ = *P*_*0*_*e*^*γAd*^ and *P*_*s*_ = *P*_*0*_*e*^*γAs*^, where *A*_*d*_ denotes the end-diastolic arterial cross-sectional area, and *A*_*s*_ denotes the end-systolic arterial cross-sectional area, further described as ${\rm{A}}\left({\rm{t}}\right)=\frac{{\rm{\pi }}{{\rm{d}}}^{2}(t)}{4}$.

3 $$\alpha =\frac{{A}_{d}{\text{ln}}\left(\frac{{p}_{d}}{{p}_{s}}\right)}{{A}_{d}-{A}_{s}}$$

4 $$p\left(t\right)={p}_{d}{e}^{\alpha (\frac{A\left(t\right)}{{A}_{d}}-1)}$$

Figure 14b shows the reconstructed pressure waveform derived from the UTA measurements overlaid with the reference data from the commercial pressure sensor. Although this is a simplified model, it has been successfully used in previous ultrasound-based blood pressure estimation studies, as mentioned by Peng et al.^39^. The UTA thus yielded a systolic pressure of 149 mmHg and a diastolic pressure of 13.5 mmHg, closely matching the sensor values of 153 mmHg and 11.2 mmHg, respectively. The resulting errors—4.0 mmHg (systolic) and 2.3 mmHg (diastolic)—demonstrate the feasibility of real-time pressure estimation using array-based ultrasound sensing. The summary statistics for multiple cycles are presented in Table 4.

**Table 4:** Trials-based measurement of the dynamic blood pressure across the phantom vessel target location # 1

Trial attempt #	Pulsatile parameters	Diastolic diameter (mm)^a^	Systolic diameter (mm)^a^
1	9 mL/80 Strokes/min	2.3	2.74
2		2.3	2.73
3		2.2	2.76

^a^Average of five samples

Figures S11 and S12 illustrate the vessel diameter changes between systolic and diastolic states under pulsatile pumping conditions. The pump stroke volume was sequentially varied from 3 mL to 6 mL and then to 9 mL, producing progressively larger pulsatile amplitudes. At each pumping condition, the phantom vessel diameter expanded during systole and contracted during diastole, with the magnitude of variation scaling directly with pump stroke volume. Using time-of-flight analysis, these diameter fluctuations were extracted from echoes of the proximal and distal vessel walls. The measured diameters were obtained; while these could be converted to pressure values using a pressure–diameter relationship, in this study they were analyzed to see the minimum and maximum deformation of the phantom vessel. The detailed comparison of measured diameters and converted pressures against reference values is summarized in Table 5, confirming that the flexible UTA provides accurate tracking of vessel compliance across different pumping conditions.

**Table 5:** Trials-based measurement of the dynamic blood pressure across the phantom vessel target location # 1 for 3 ml, 6 ml, and 9 ml

Trial attempt #	Pulsatile volume (ml)	Diastolic diameter (mm)^a^	Systolic diameter (mm)^a^
4	3	2.15	2.25
5	6	2.34	2.43
3	9	2.20	2.76

^a^Average of five samples

Table 6 presents a comparative performance analysis. Peng et al.^39^ reported exceptional accuracy with a monolithic 6 mm × 6 mm PZT 1-3 element, achieving errors within ±0.8 mmHg. However, the tradeoff involves reduced flexibility and larger device dimensions. In contrast, our miniaturized 5 × 4 array, composed of 1.2 mm × 1.2 mm PMN-PT elements, demonstrated high measurement fidelity despite the increased variability inherent in flexible systems. Furthermore, unlike monolithic patches, the proposed UTA architecture enables spatially distributed acquisition and redundancy.

**Table 6:** Comparison of the reference work with the present work

Reference #	Device type	Material	Systolic BP error	Diastolic BP error	Key notes
Peng et al. 2021 (ref. ^39^)	Single transducer with PDMS packaging	Thin film 1-3 PZT composite	±0.87 mmHg	±0.78 mmHg	High-fidelity waveform with distinct systolic/diastolic peaks
Liu et al. 2024 (ref. ^40^)	5 × 5 element array optimally designed with PDMS packaging	Thin film PZT-5H	**Relative error (Mean relative error—MRE)** with commercial device: 6.14%, 5.89%, 6.15%, and 7.00%		Phased-array focused emission and real-time detection of wall displacement to reconstruct BP waveform
Wang et al. 2018 (ref. ^29^)	4 × 5 element array designed with elastomer packaging	Thin film 1-3 PZT composite	Up to 2 mmHg		Echo-based wall displacement and estimates BP from vessel wall motion
This study	5 × 4 element UTA with low temperature eutectic solder and parylene coating	Thin film 1-3 PMN-PT composite	Average 4 mmHg	Average 2.3 mmHg	Low-temp Sn-Bi, solder bonding preserves piezo properties and TOF-based vessel diameter tracking

The relative errors reported by Liu et al.^40^ and Wang et al.^29^ ranged from 2 to 8 mmHg under varying in vitro and phantom conditions. Although slightly higher, our results remain within the ±5 mmHg mean error limit recommended by the Advancement of Medical Instrumentation (AAMI) standard for clinical-grade devices^41^, underscoring the practical relevance of the proposed platform. Residual discrepancies have been attributed to multiple factors: mechanical compliance variability of the phantom vessel, acoustic impedance mismatch due to the absence of matching layers, and limited spatial coherence in array-wide signal processing. Future refinements, particularly in phantom elasticity control, element-level focusing, and adaptive beamforming, are expected to enhance the fidelity of waveform reconstruction. A limitation of this study is that validation was restricted to vascular phantom experiments, without in vivo or on-body testing. While the phantom model allowed controlled evaluation of vessel diameter tracking and pressure waveform estimation, future work will focus on pilot human studies to demonstrate the translational potential ability of the sensor. On-body testing at superficial arteries such as the radial or brachial artery will provide essential insights into signal fidelity, motion robustness, and long-term wearability in real physiological environments.

## Conclusion

This paper presented the design, fabrication, and comprehensive evaluation of a flexible 5 × 4 ultrasonic transducer array (UTA) based on PMN-PT 1-3 composite elements engineered for continuous, noninvasive blood pressure monitoring. By leveraging a dual-sided eutectic solder bonding process, the device achieved low-temperature integration that preserves the piezoelectric and electromechanical integrity of the PMN-PT elements while ensuring mechanical compliance and electrical connections suitable for skin-mounted applications. Detailed electrical and acoustic characterization demonstrated a resonant frequency of approximately 6.0 MHz, a −3 dB bandwidth of 38%, and an impressive 45° acceptance angle were achieved without the use of acoustic matching or backing layers, thus simplifying fabrication while maintaining high signal fidelity.

In vitro validation using vascular phantoms confirmed the capability of the array to dynamically track vessel-diameter changes and accurately reconstruct systolic and diastolic pressure waveforms with measurement deviations within clinically acceptable margins. In addition to its immediate utility in wearable cardiovascular diagnostics, this study demonstrated a scalable approach for integrating high-performance piezoelectric materials into flexible platforms, thereby opening new avenues for next-generation wearable ultrasound systems. Future work will focus on optimizing beamforming strategies, introducing advanced acoustic coupling layers, and pursuing in vivo evaluations, moving closer to the realization of continuous point-of-care hemodynamic monitoring in both clinical and home environments.

## Supplementary information

Supplementary File

Movie S1

Movie S2

## Data Availability

The main data had been provided in the article and the Supplementary Material. Any other raw/processed data required to reproduce the findings of this study are available from the corresponding authors upon request.
