# Supplementary material for: Skin-conformal PMN-PT ultrasonic sensor for cuffless blood pressure sensing via eutectic solder integration
Source: Microsyst Nanoeng. 2026 Jan 1;12:6. doi: 10.1038/s41378-025-01110-2 (PMC12756301; doi:10.1038/s41378-025-01110-2)
Supplement: Supplementary file 1 — Supplementary File [file 41378_2025_1110_MOESM1_ESM.pdf]

Supplementary Material

Skin-Conformal PMN-PT Ultrasonic Sensor for Cuffless Blood Pressure Sensing via Eutectic Solder Integration

Syed Turab Haider Zaidi[1,3,†], Dong Hun Kim[2,†], Muhammad Ali Shah[1], Young Jin Lee[1], Byung Chul Lee[2,3,4,*], and Shin Hur[1,3,*]

[1]Department of Bionic Machinery, Korea Institute of Machinery and Materials, Daejeon, 34103, South Korea

[2]Bionics Research Centers, Korea Institute of Science and Technology, 02792, Seoul, South Korea

[3]University of Science and Technology, 34113, Daejeon, Korea

[4]KHU-KIST Department of Converging Science and Technology, Kyung Hee University, 02447, Seoul, South Korea

† Contributed equally

[*]Correspondence: Shin Hur (shur@kimm.re.kr); Byung Chul Lee (bclee@kist.re.kr)

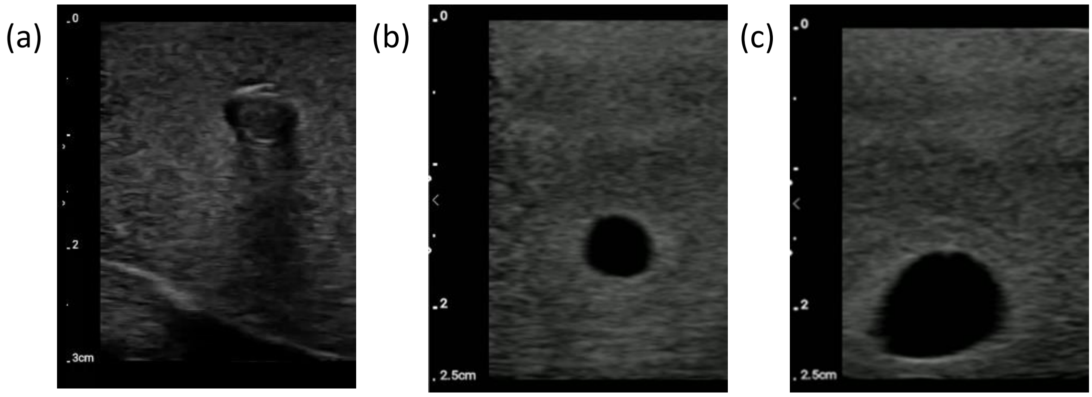

Fig. S1. Measurement of the phantom vessel diameter using the commercial L12-4V probe at (a) Location 1, (b) Location 2, and (c) Location 3

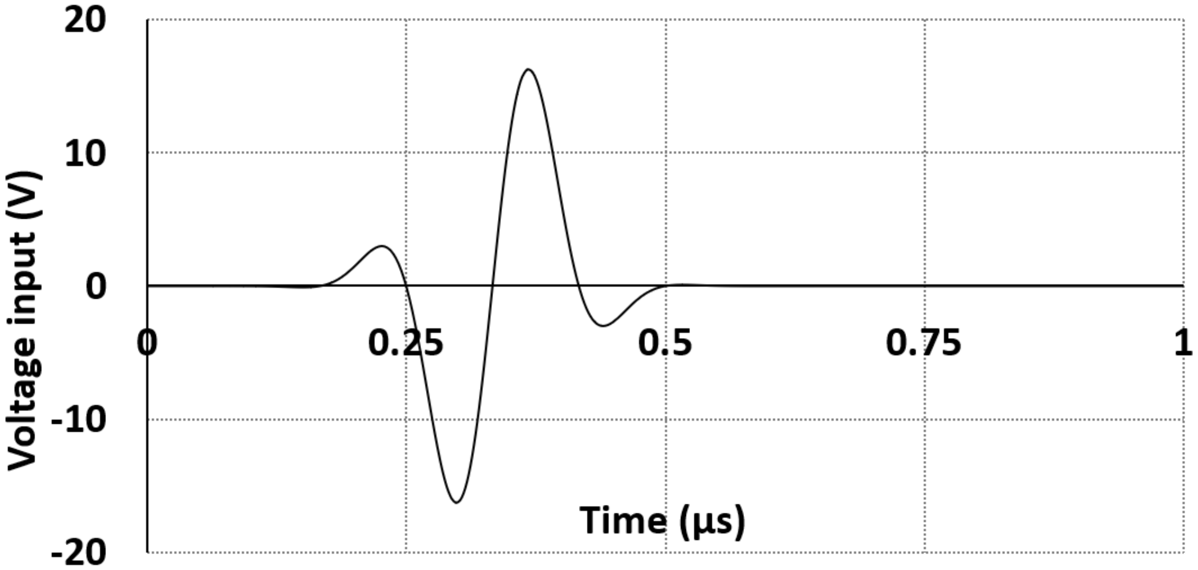

Fig. S2 Applied pulse echo input signal in COMSOL FEM model

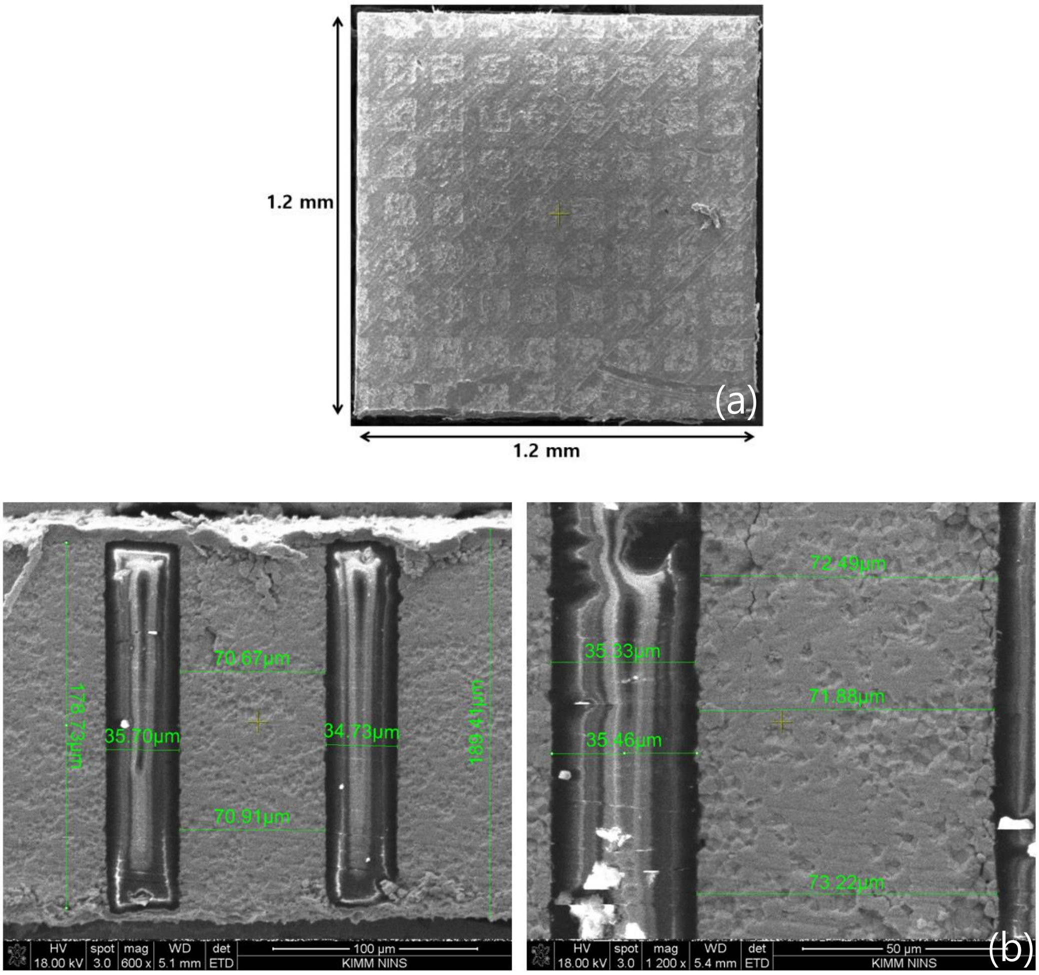

Fig. S3. (a) SEM Image of top view of the composite, (b) Cross-section view and zoom-in view of the 1-3 composite

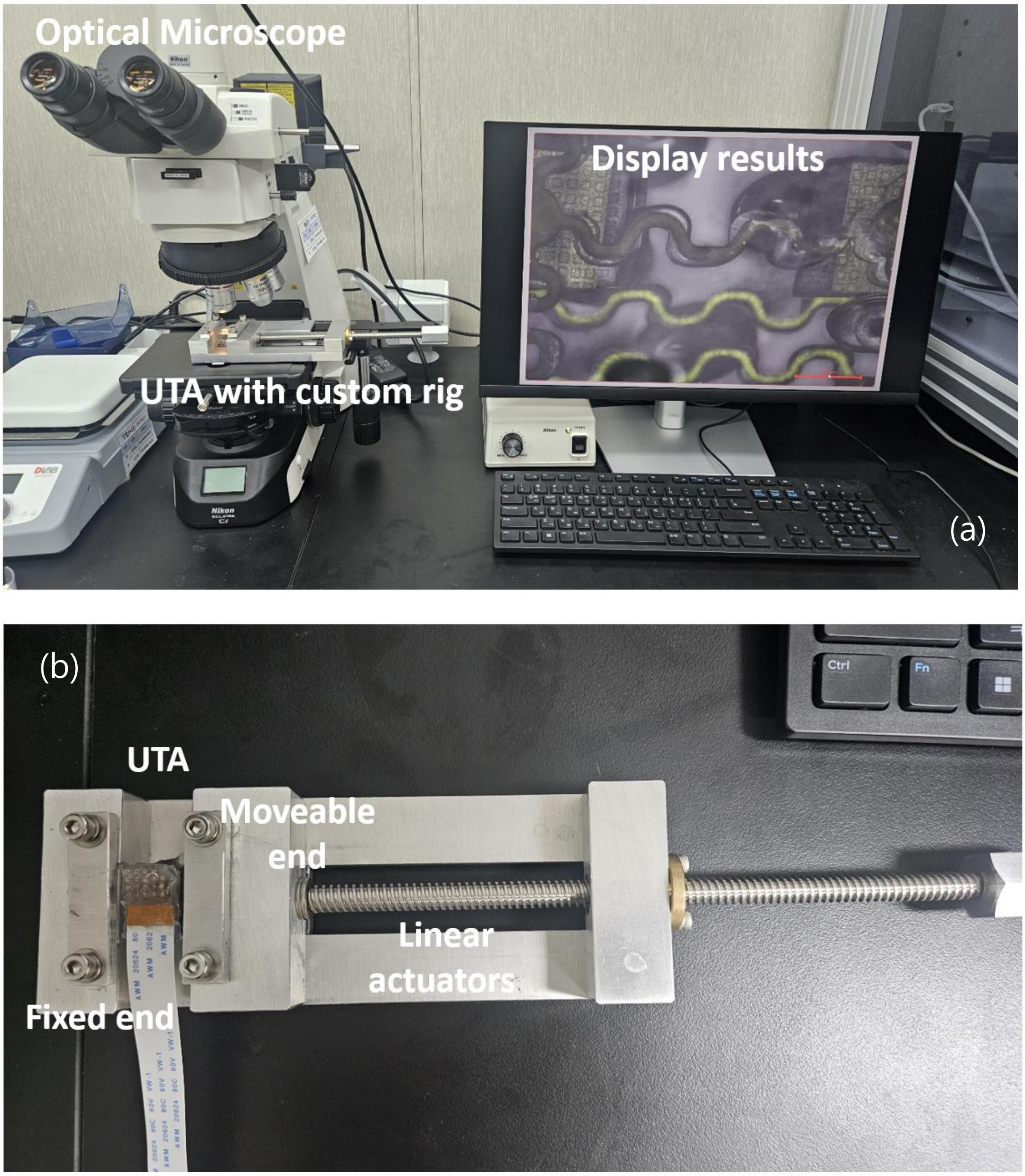
Optical Microscope
Display results
UTA with custom rig
(a)
(b)
Ctrl
Fn
UTA
Moveable end
Linear actuators
Fixed end

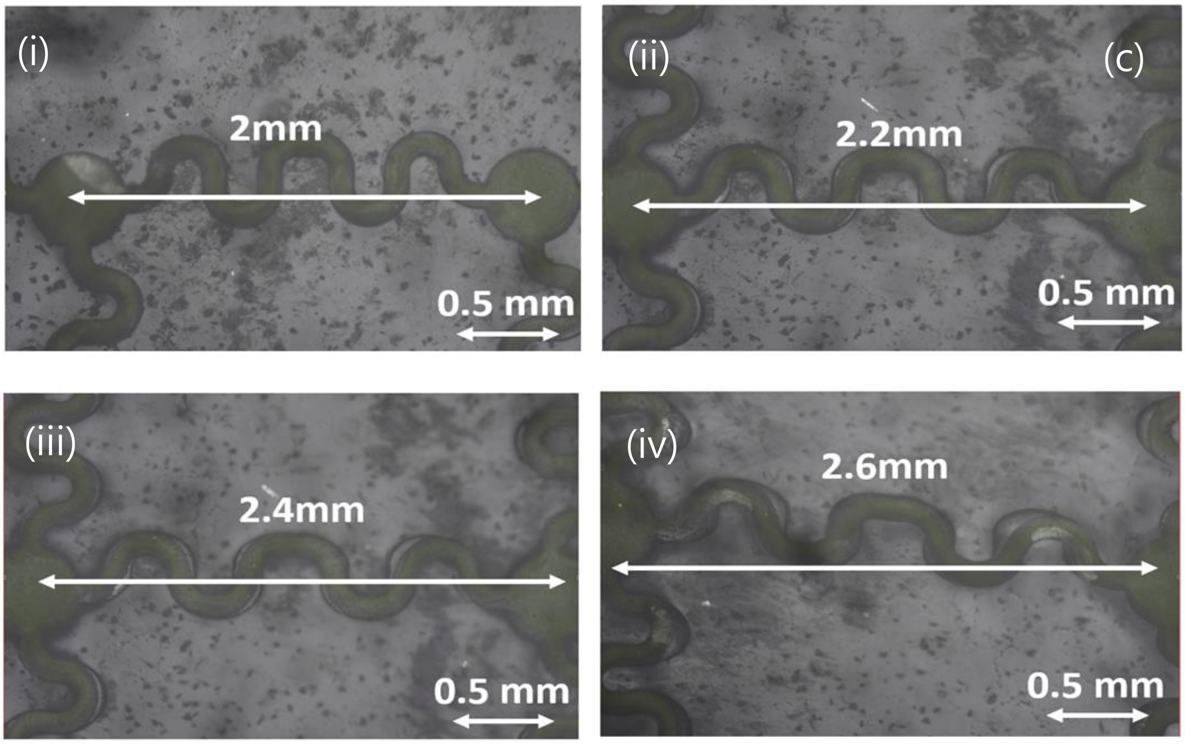

Fig. S4. (a) Test setup of the strain measurement, **(b)** Illustrations of the custom linear actuator **(c)** tensile loading on serpentine electrode from (i) 0 mm, (ii) 0.2 mm, (iii) 0.4 mm, (iv) 0.6 mm

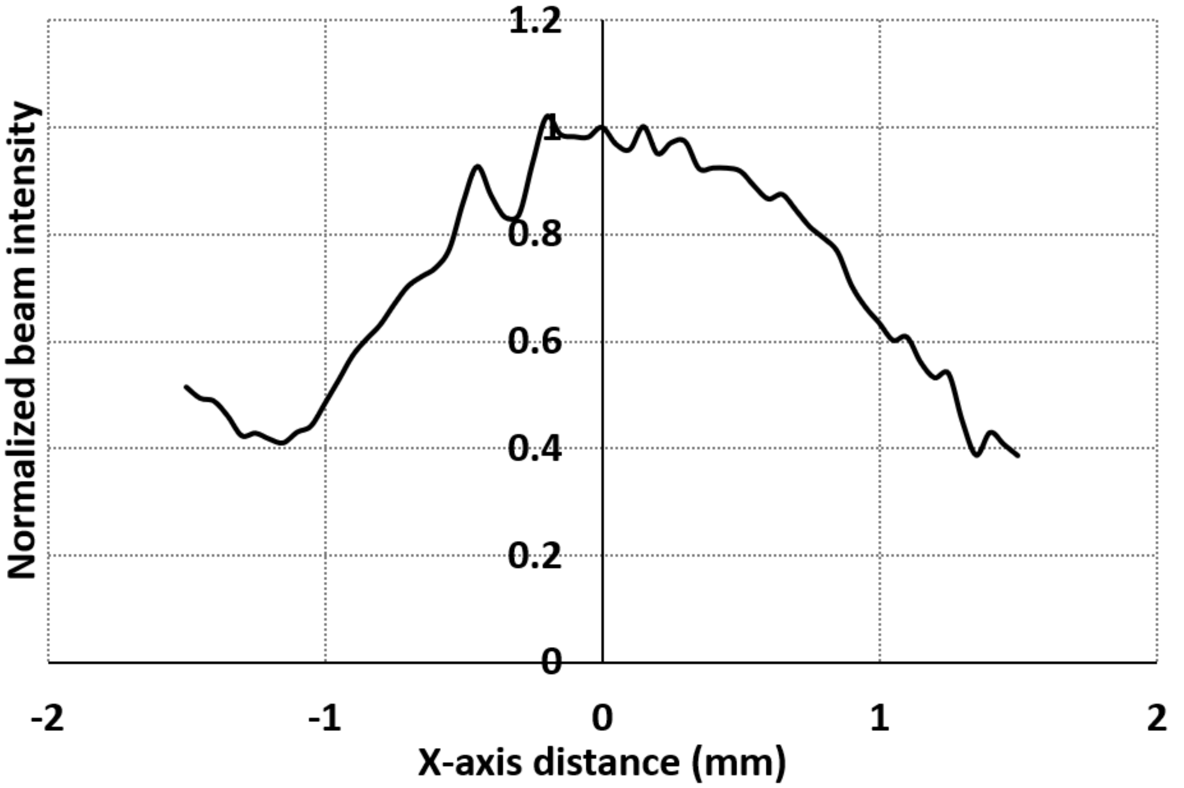

Fig. S5 Measurement of the UTA beam directivity measured by the hydrophone across the X axis at a depth of 15 mm.

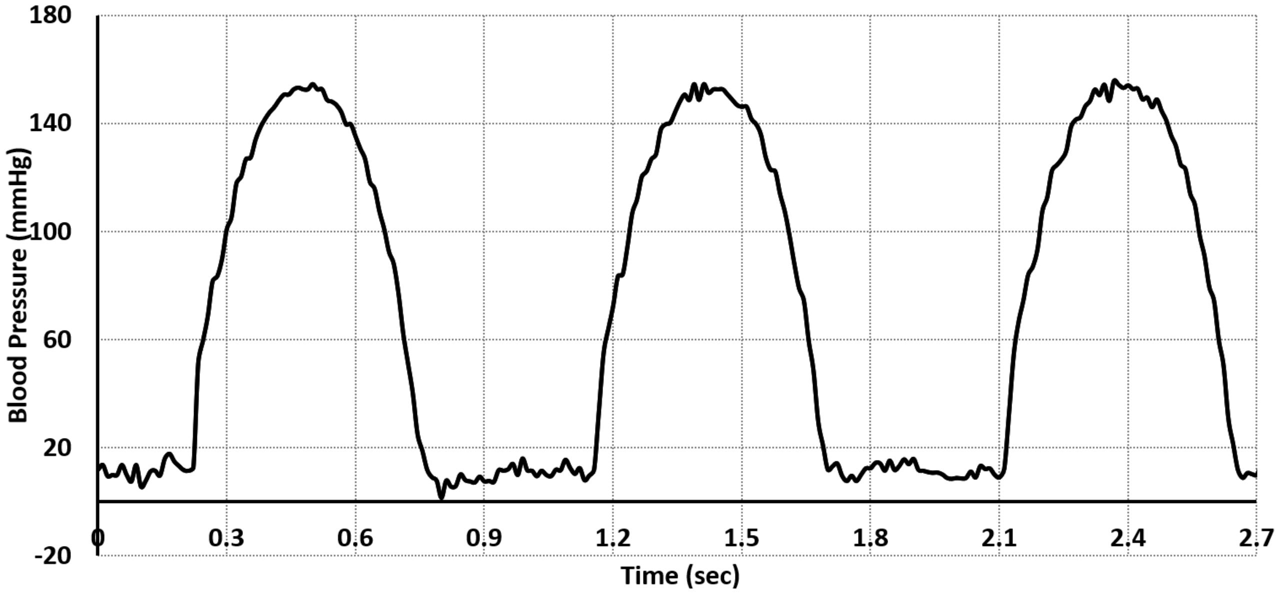

Fig. S6 Measurement of the pressure of the liquid flowing inside the phantom tube and achieved by the use of pressure sensor model 33A-005G-210

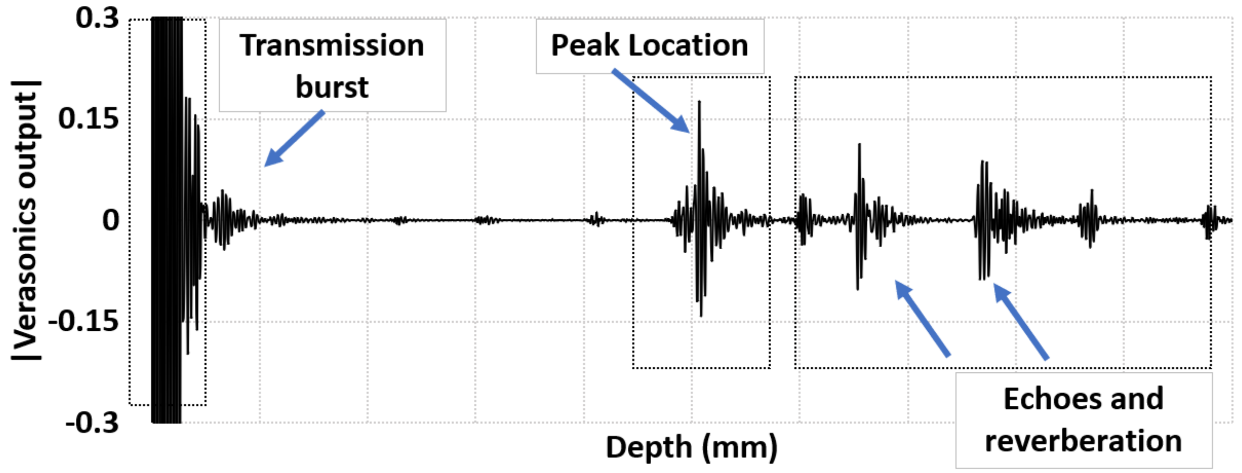

Fig. S7 Example of an RF-Output of the 1D array broken down in specific regions

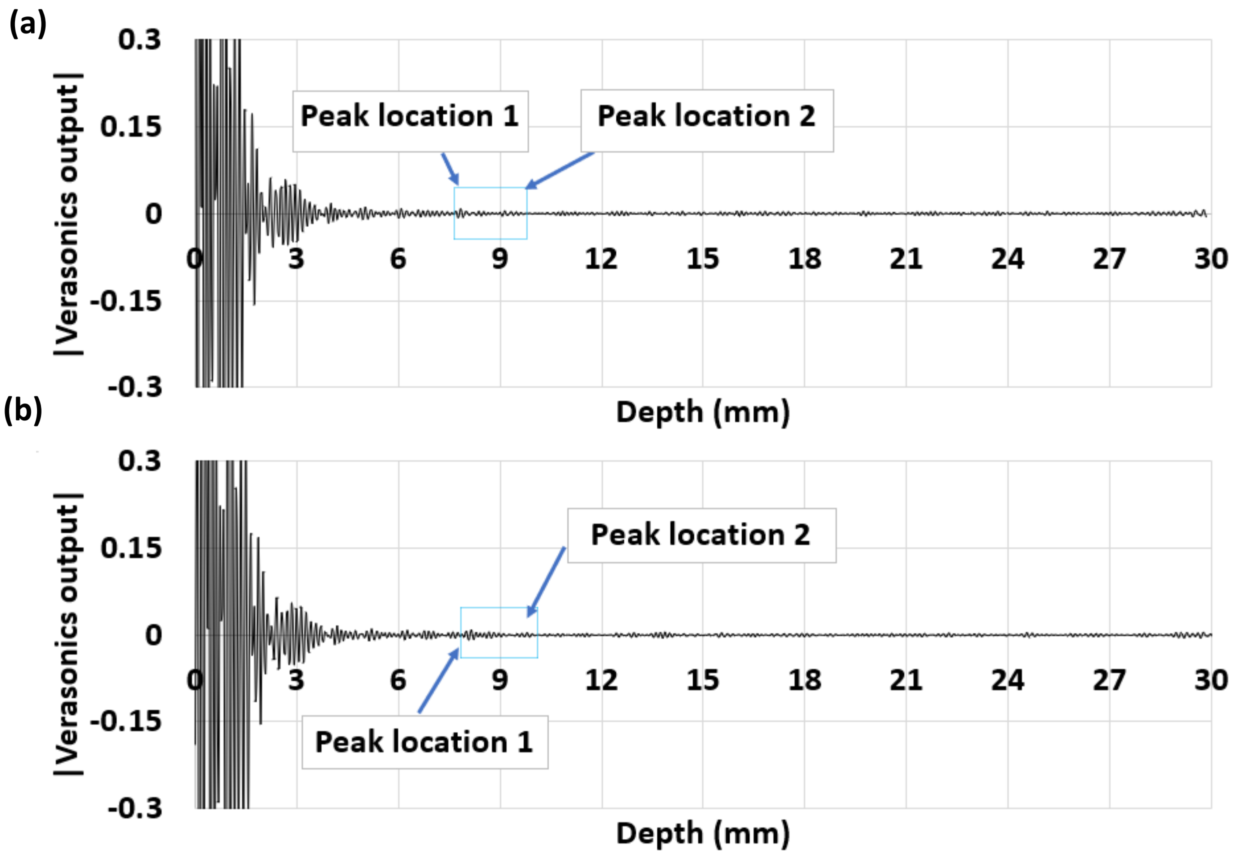

Fig. S8 RF-scan obtained from an individual transducer element acquired at different frames from a UTA element

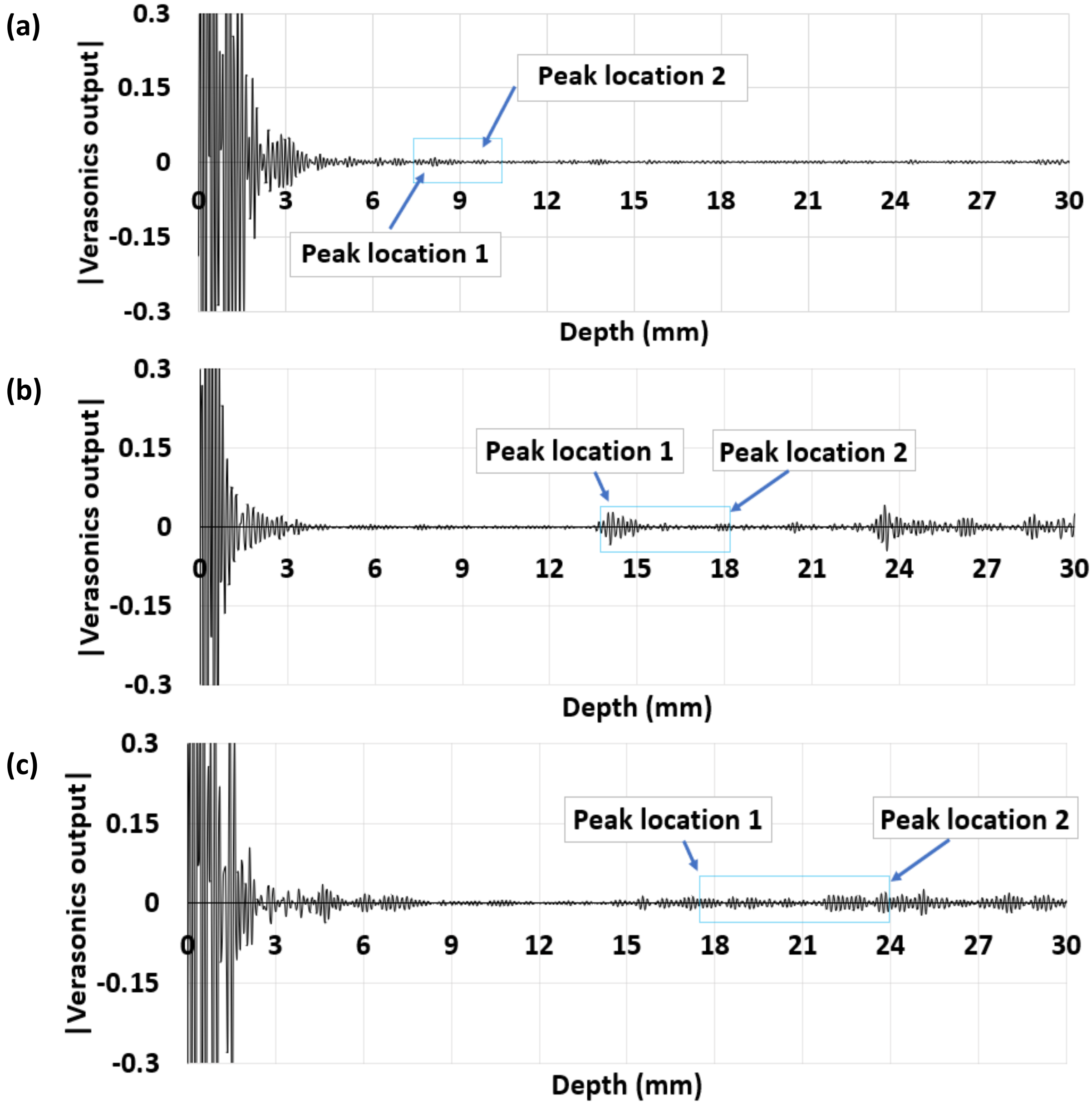

Fig. S9 RF-Outputs of different average measurement from the UTA for (a) – (c) Location 1 to Location 3

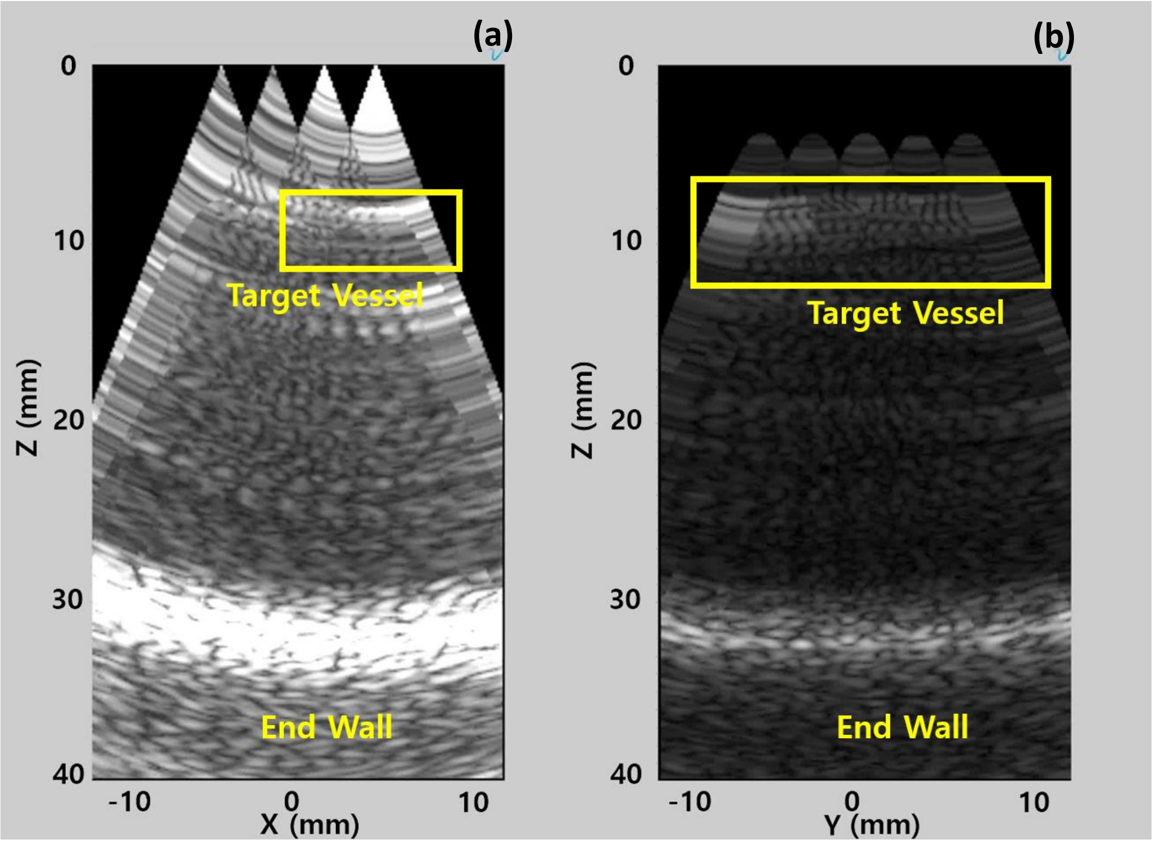

Fig. S10 B-Mode Imaging obtained from Verasonics with un focused 1D array of elements. (a) Cross section identification of Target Location # 1, (b) Longitudinal axis of Target Location # 1

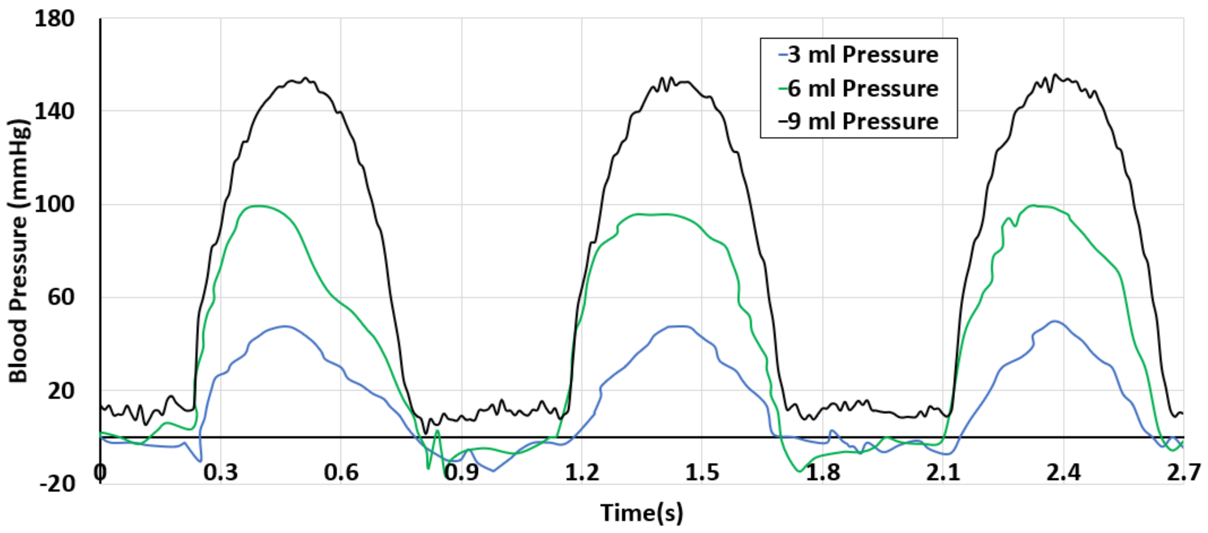

Fig. S11 Comparison of blood pressure measure for target vessel # 1 with flow volume variation from 3 ml, 6 ml, and 9ml

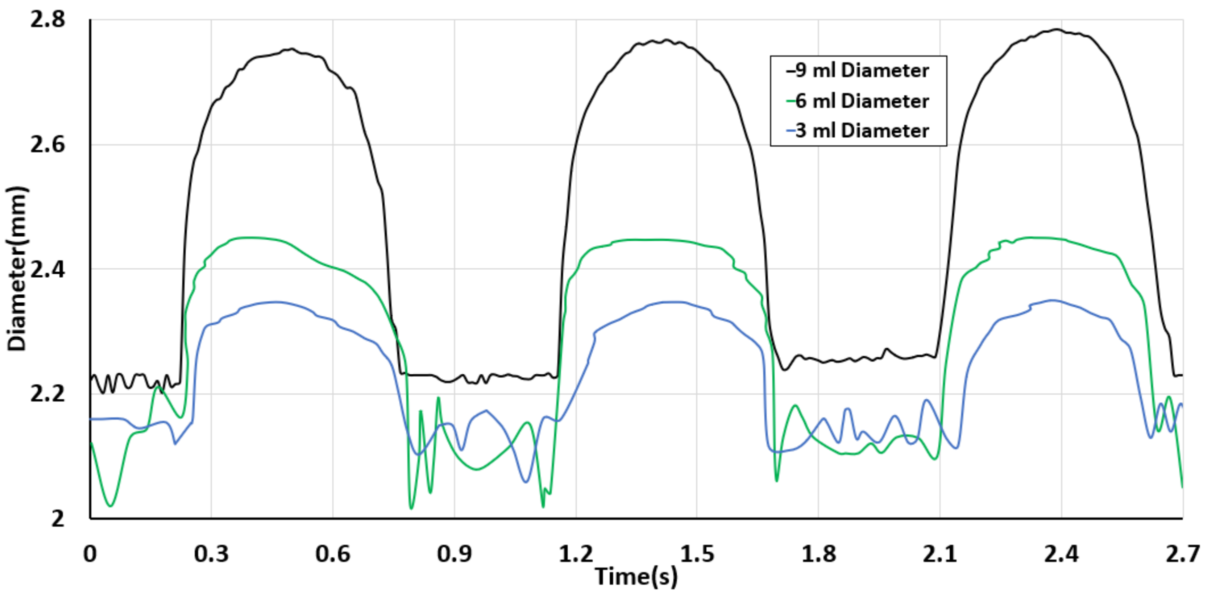

Fig. S12 Comparison of diameter for target vessel # 1 with flow volume variation from 3 ml, 6 ml, and 9ml
